# Rheumatoid Arthritis Mimickers Presenting With Polyarticular Arthritis: A Case Series Highlighting Diagnostic Pitfalls

**DOI:** 10.7759/cureus.114738

**Published:** 2026-08-18

**Authors:** Ibrahim Saleh, Amjad Alkadi, Emad Wissa, Saleh Salman, Mostafa Abdelaty

**Affiliations:** 1 Rheumatology, Kuwait Ministry of Health, Kuwait City, KWT; 2 Rheumatology, Al-Sabah Hospital, Kuwait Ministry of Health, Kuwait City, KWT; 3 Internal Medicine, Al-Sabah Hospital, Kuwait City, KWT; 4 Medicine/Cardiology, Aleppo University Hospital, Aleppo, SYR; 5 Respiratory Medicine, Al-Sabah Hospital, Kuwait City, KWT

**Keywords:** crystal arthropathy, paraneoplastic arthritis, polyarticular arthritis, rheumatoid arthritis, rs3pe

## Abstract

Rheumatoid arthritis (RA) is the most common chronic inflammatory arthritis and a leading cause of disability worldwide. However, several diseases may mimic RA, particularly in patients presenting with polyarticular pain or seronegative inflammatory arthritis. Misdiagnosis may result in inappropriate immunosuppressive therapy and delayed treatment of the underlying disease. Recognizing these mimickers remains a critical challenge in rheumatology practice.

We conducted a retrospective case series of patients evaluated at a tertiary rheumatology center between January 2021 and December 2025. Patients presenting with clinical features suggestive of RA but subsequently diagnosed with alternative conditions were included. Clinical presentation, laboratory findings, imaging studies, diagnostic reasoning, and treatment outcomes were reviewed.

Five patients initially suspected to have RA were ultimately diagnosed with alternative conditions. These included ferric carboxymaltose-induced hypophosphatemic osteomalacia, calcific tendinitis, polyarticular gout, remitting seronegative symmetrical synovitis with pitting edema (RS3PE) associated with malignancy, and carcinomatous polyarthritis secondary to colon adenocarcinoma. Several diagnostic clues including atypical joint involvement, abnormal laboratory findings, imaging abnormalities, and systemic symptoms helped establish the correct diagnoses.

RA mimickers represent an important diagnostic challenge in rheumatology practice. Crystal arthropathies, metabolic bone diseases, calcific tendinitis, and paraneoplastic syndromes may closely resemble RA. Awareness of atypical clinical features and recognition of diagnostic red flags are essential for accurate diagnosis. Early identification of these conditions allows appropriate treatment and prevents unnecessary exposure to immunosuppressive therapies.

## Introduction

Rheumatoid arthritis (RA) is a chronic systemic autoimmune disease characterized by persistent synovitis, progressive joint destruction, and various extra-articular manifestations. The disease affects approximately 0.5%-1% of the adult population and represents one of the most common causes of inflammatory polyarthritis worldwide. Early recognition and prompt initiation of disease-modifying antirheumatic drugs (DMARDs) are essential to prevent irreversible joint damage and functional disability [1].

Despite advances in classification criteria and diagnostic tools, the diagnosis of RA remains primarily clinical and can be challenging in early disease or atypical presentations. The 2010 American College of Rheumatology/European League Against Rheumatism (ACR/EULAR) classification criteria improved sensitivity for early RA but may also lead to misclassification when applied to patients with other inflammatory or metabolic disorders that resemble RA. Consequently, several diseases may initially present with symptoms mimicking RA [2].

A wide spectrum of conditions can mimic RA, including crystal-induced arthropathies such as gout and calcium hydroxyapatite deposition disease, metabolic bone disorders such as osteomalacia, paraneoplastic syndromes including carcinomatous polyarthritis, and drug-related complications. These conditions may present with polyarticular pain, elevated inflammatory markers, or radiographic findings similar to RA, leading to diagnostic confusion [3].

Paraneoplastic rheumatic syndromes are particularly important RA mimickers. Carcinomatous polyarthritis and remitting seronegative symmetrical synovitis with pitting edema (RS3PE) may present with inflammatory arthritis resembling RA and may precede the diagnosis of malignancy by months or even years. Early recognition of these syndromes is crucial because treatment of the underlying malignancy often results in resolution of the rheumatic manifestations [4,5].

Similarly, metabolic and drug-induced conditions may present with musculoskeletal symptoms mimicking inflammatory arthritis. Hypophosphatemic osteomalacia associated with ferric carboxymaltose therapy has increasingly been recognized as a cause of bone pain, muscle weakness, and multiple stress fractures. This condition results from fibroblast growth factor-23 (FGF-23)-mediated renal phosphate wasting and impaired bone mineralization [6,7].

Crystal-induced arthropathies may also present with polyarticular involvement resembling RA. Although gout classically presents as podagra, many patients-particularly elderly individuals or those with chronic disease-may develop polyarticular gout mimicking inflammatory polyarthritis. Calcium hydroxyapatite deposition disease, particularly calcific tendinitis, may also present with acute severe joint pain and restricted movement that can mimic inflammatory arthritis [8,9].

Failure to recognize these mimickers may expose patients to unnecessary immunosuppressive therapy and delay appropriate management of the underlying condition. Therefore, identifying clinical red flags that suggest alternative diagnoses is an essential aspect of rheumatology practice.

In this report, we present a case series of five patients initially suspected to have RA but subsequently diagnosed with alternative conditions. These cases illustrate important diagnostic pitfalls and highlight the importance of maintaining a broad differential diagnosis when evaluating patients with polyarticular symptoms.

This case series highlights a spectrum of clinically important RA mimickers encountered in real-world rheumatology practice. By presenting these cases together, we aim to emphasize key diagnostic clues and clinical red flags that may help clinicians differentiate RA from other conditions presenting with polyarticular arthritis.

## Case presentation

We present a retrospective case series of five patients evaluated at a tertiary rheumatology center who were initially suspected to have RA but were subsequently diagnosed with alternative conditions mimicking RA. Clinical presentation, laboratory investigations, imaging findings, diagnostic workup, treatment, and outcomes were reviewed using anonymized medical records in accordance with the principles of the Declaration of Helsinki.

Case 1: ferric carboxymaltose-induced hypophosphatemic osteomalacia

A 55-year-old woman with long-standing seropositive RA presented with worsening generalized body pain, fatigue, and diffuse musculoskeletal discomfort, initially raising concern for an RA flare. Her RA had been well controlled on tocilizumab therapy following failure of conventional DMARDs. The patient also had a history of iron deficiency anemia treated with intravenous ferric carboxymaltose.

On clinical examination, no active synovitis was detected. Tenderness was present in multiple fibromyalgia tender points, with preserved range of motion in the joints. Laboratory investigations demonstrated normal inflammatory markers, making an RA flare unlikely.

Further metabolic evaluation revealed hypophosphatemia, elevated alkaline phosphatase, and increased urinary phosphate excretion. Parathyroid hormone levels were mildly elevated, while serum calcium remained within the low-normal range. Bone scintigraphy demonstrated increased uptake in the shoulders and clavicles consistent with metabolic bone disease.

Based on these findings, the diagnosis of ferric carboxymaltose-induced hypophosphatemic osteomalacia was established. Ferric carboxymaltose therapy was discontinued, and the patient was treated with phosphate and vitamin D supplementation. Clinical symptoms gradually improved following correction of phosphate levels.

Ferric carboxymaltose is increasingly recognized as a cause of FGF-23-mediated renal phosphate wasting leading to osteomalacia. Patients may present with bone pain, muscle weakness, and stress fractures that can mimic inflammatory arthritis [8,9].

**Figure 1:**
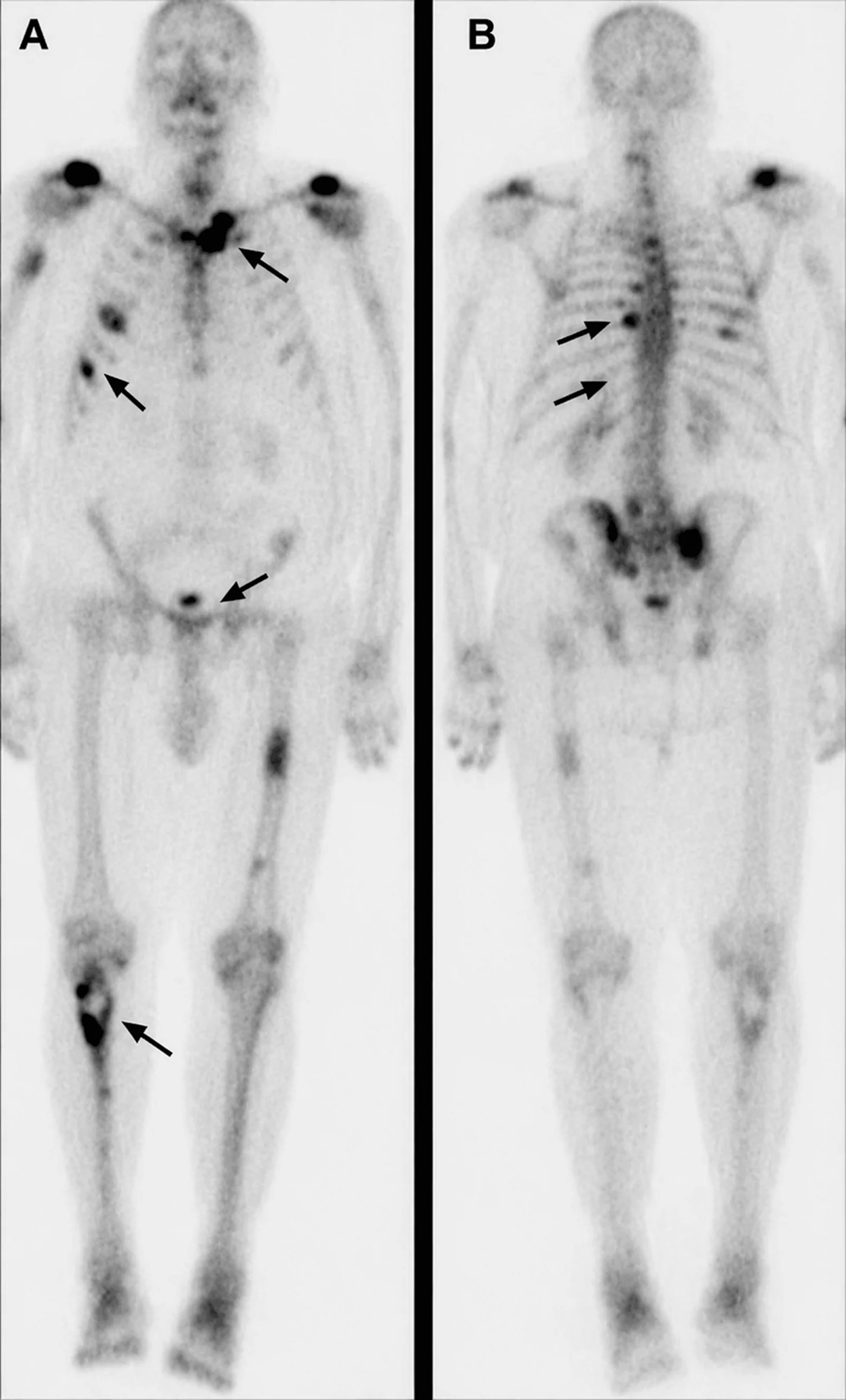
Whole-body bone scintigraphy in ferric carboxymaltose-induced hypophosphatemic osteomalacia. (A) Anterior view and (B) posterior view demonstrating multiple areas of increased tracer uptake involving the shoulders, clavicles, ribs, pelvis, and long bones, consistent with metabolic bone disease secondary to hypophosphatemia.

The characteristic biochemical abnormalities observed in this patient are summarized in Table 1, and bone scintigraphy findings are shown in Figure 1.

**Table 1:** Biochemical profile in ferric carboxymaltose-induced hypophosphatemic osteomalacia. - indicates data not available or not applicable. PTH: parathyroid hormone

Parameter	Result	Reference range
Serum phosphate	0.3 mmol/L	0.8-1.5 mmol/L
Calcium	2.1 mmol/L	2.1-2.6 mmol/L
Alkaline phosphatase	150 U/L	<120 U/L
Vitamin D	65 nmol/L	>50 nmol/L
PTH	75 pg/mL	10-65 pg/mL
Urinary phosphate	>20%	-

Case 2: calcific tendinitis mimicking RA

A 55-year-old woman presented with recurrent shoulder pain associated with hand and knee pain. She had previously been diagnosed with seropositive RA, primarily based on mildly elevated rheumatoid factor and inflammatory markers in the setting of polyarticular joint pain.

Physical examination revealed marked restriction of active and passive movement of the right shoulder. However, there was no objective synovitis in the small joints of the hands.

Radiographs of the shoulder revealed calcific deposits near the rotator cuff insertion consistent with calcific tendinitis caused by hydroxyapatite crystal deposition. The patient was treated with a local corticosteroid injection to the affected shoulder and intermittent colchicine therapy. Symptoms resolved completely, and full range of motion was restored.

**Figure 2:**
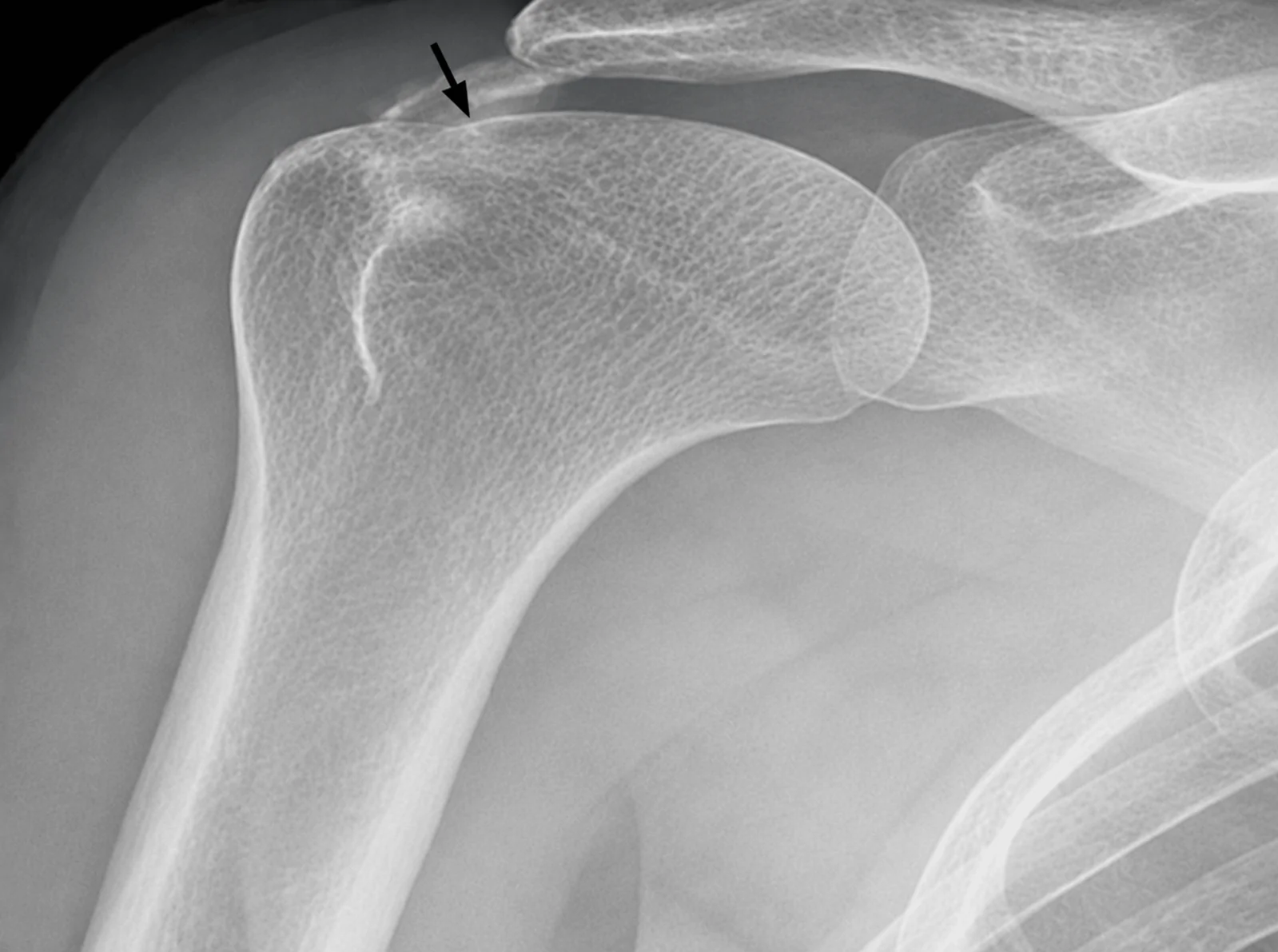
Shoulder radiograph demonstrating calcific tendinitis. Plain radiograph showing amorphous calcific deposits adjacent to the rotator cuff tendon insertion consistent with hydroxyapatite crystal deposition.

Calcific tendinitis is a self-limiting inflammatory condition caused by deposition of calcium hydroxyapatite crystals in peri-tendinous tissues. The shoulder, particularly the supraspinatus tendon, is the most commonly affected site. Acute presentations may mimic inflammatory arthritis or septic arthritis [9]. A shoulder radiograph is shown in Figure 2.

Case 3: polyarticular gout mimicking RA

A 45-year-old man presented with a five-year history of episodic joint pain involving the hands, knees, shoulders, and feet. Initially, the symptoms occurred in attacks but later progressed to persistent joint pain and swelling, raising suspicion for RA due to the chronic polyarticular pattern.

Musculoskeletal examination demonstrated synovitis in the metacarpophalangeal and proximal interphalangeal joints. Laboratory tests revealed elevated inflammatory markers and hyperuricemia.

Radiographs of the hands demonstrated findings compatible with gouty arthropathy. The patient was diagnosed with polyarticular gout, an atypical presentation that can resemble RA.

Treatment with colchicine and intra-articular corticosteroid injection resulted in significant improvement. Urate-lowering therapy with febuxostat was initiated, and the patient remained in remission during follow-up.

**Figure 3:**
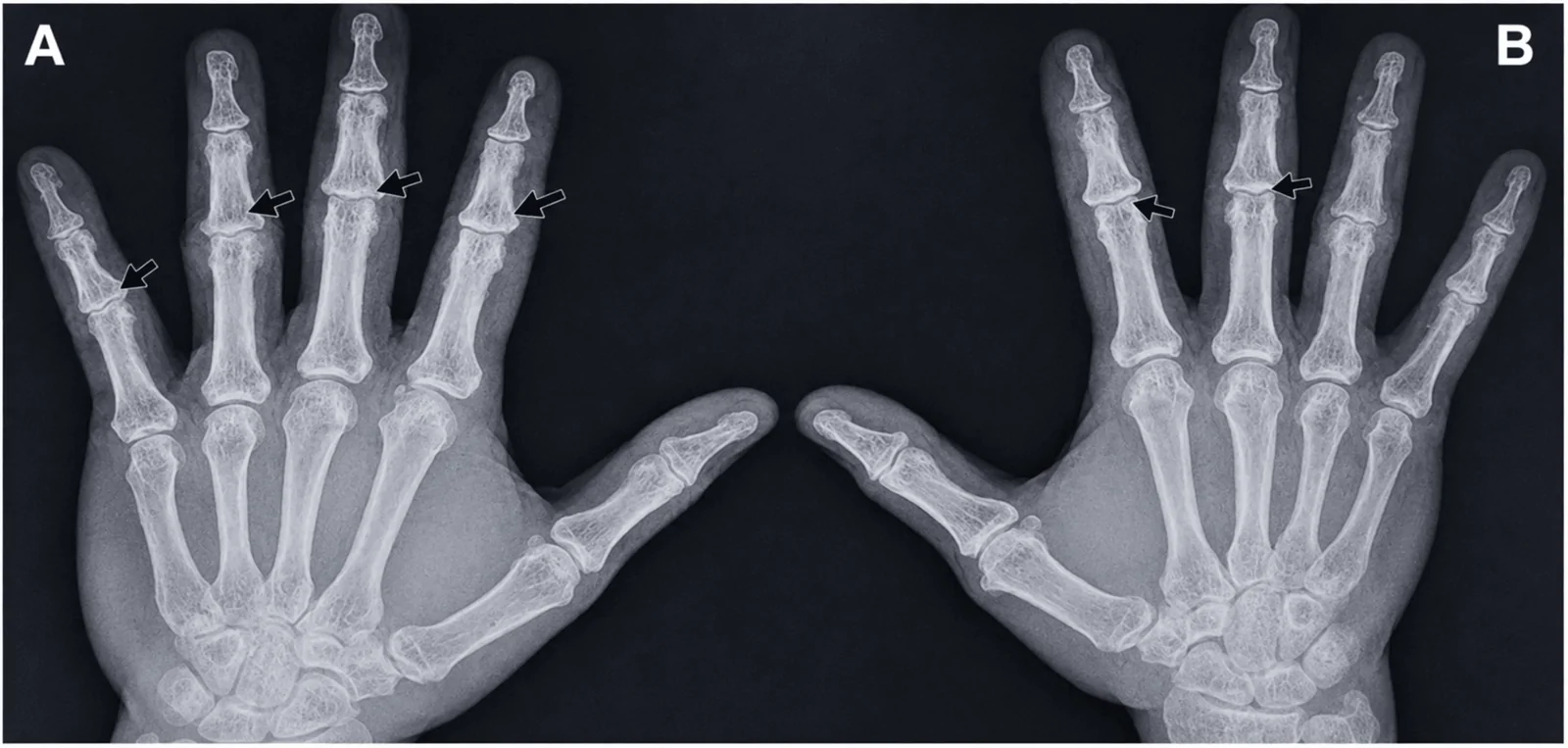
Bilateral hand radiographs demonstrating chronic gouty arthropathy. (A) Left hand showing multiple punched-out erosions with overhanging edges. (B) Right hand demonstrating similar erosive changes consistent with chronic gout.

Although gout classically presents as podagra, polyarticular involvement is increasingly recognized, particularly in chronic disease. Such presentations may mimic RA both clinically and radiographically [8]. Hand radiographic findings are shown in Figure 3.

Case 4: RS3PE associated with malignancy

A 68-year-old woman presented with acute-onset polyarthritis involving the wrists, metacarpophalangeal joints, and ankles. The condition was associated with bilateral pitting edema of the hands and significant unintentional weight loss.

Laboratory investigations showed markedly elevated inflammatory markers, while rheumatoid factor and anti-cyclic citrullinated peptide (CCP) antibodies were negative. The clinical presentation was consistent with RS3PE. The patient responded dramatically to low-dose corticosteroid therapy.

However, further malignancy screening was performed due to the association of RS3PE with paraneoplastic syndromes. Imaging revealed a uterine mass consistent with an incidental uterine fibroid. Subsequent hematological evaluation demonstrated blast cells on peripheral blood smear, and bone marrow biopsy confirmed acute myeloid leukemia.

**Figure 4:**
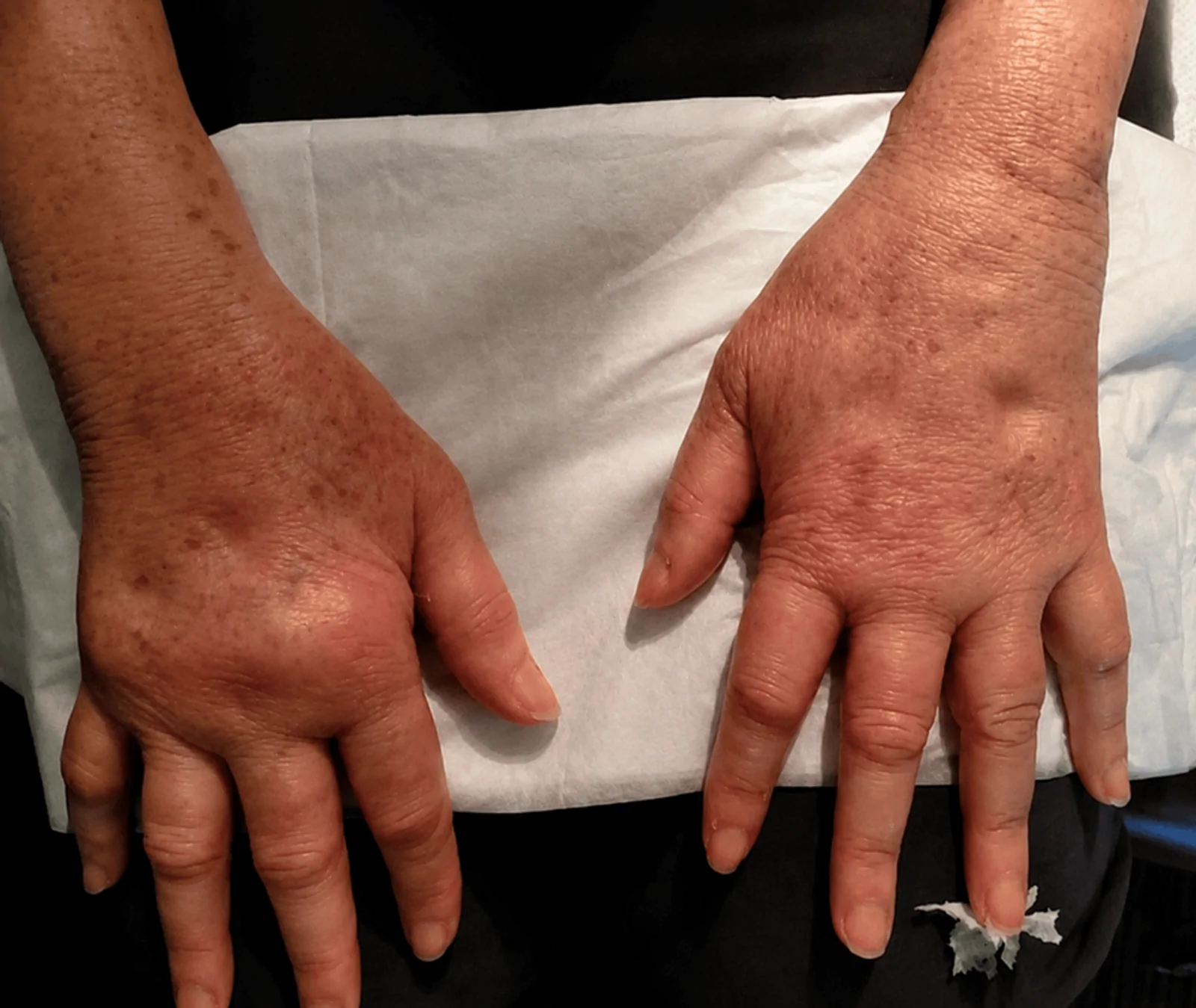
Clinical presentation of RS3PE syndrome. Bilateral pitting edema of the hands with symmetrical synovitis involving the wrists and metacarpophalangeal joints. RS3PE: remitting seronegative symmetrical synovitis with pitting edema

RS3PE is a rare inflammatory syndrome typically affecting elderly individuals and characterized by symmetrical synovitis and pitting edema of the hands. It has been associated with various malignancies, highlighting the importance of malignancy screening in these patients [4,5]. Clinical findings are demonstrated in Figure 4.

Case 5: carcinomatous polyarthritis

A 76-year-old man with previously well-controlled RA presented with new-onset polyarthritis involving the hands, knees, and ankles accompanied by markedly elevated inflammatory markers. Despite high inflammatory markers, the degree of synovitis was relatively mild. Laboratory investigations also revealed microcytic anemia.

Given the discordance between clinical findings and inflammatory markers, further evaluation was performed. Endoscopic examination revealed a large cecal mass, and biopsy confirmed colon adenocarcinoma.

The diagnosis of carcinomatous polyarthritis, a paraneoplastic rheumatic syndrome, was established. The patient was referred for oncologic management.

**Figure 5:**
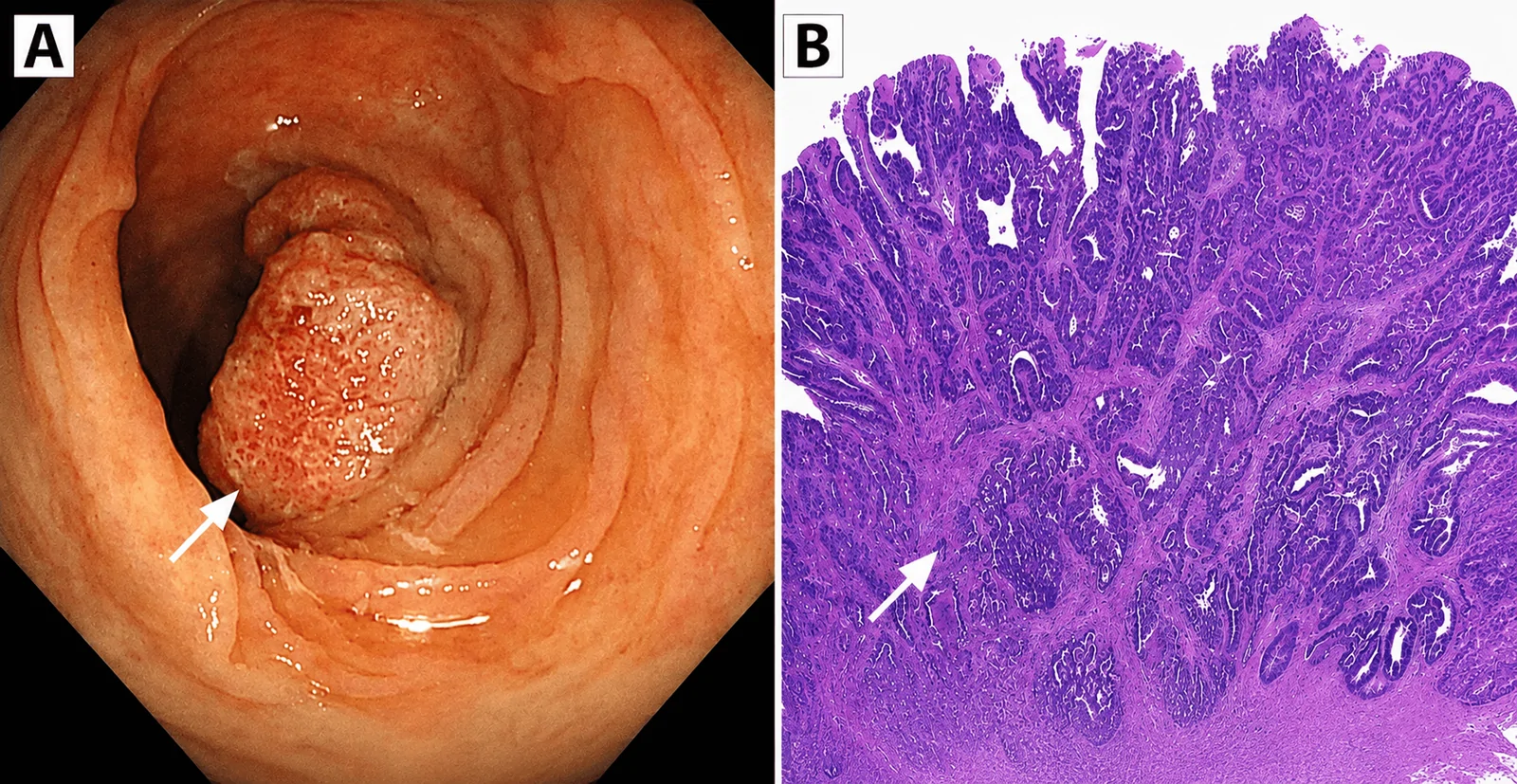
Colon adenocarcinoma associated with carcinomatous polyarthritis. (A) Colonoscopic image demonstrating a fungating cecal mass. (B) Histopathological section (H&E staining) demonstrating adenocarcinoma.

Carcinomatous polyarthritis is a rare paraneoplastic condition that may mimic RA and often precedes the diagnosis of malignancy. Recognition of atypical clinical features such as late-onset arthritis and unexplained systemic symptoms is essential [4,10]. Endoscopic and histopathological findings are shown in Figure 5.

Collectively, these five cases illustrate the broad spectrum of diseases that may mimic RA. Despite their diverse etiologies, several common clinical features and diagnostic clues facilitated the correct diagnosis. A summary of the clinical characteristics of all cases is presented in Table 2.

**Table 2:** Summary of cases. RA: rheumatoid arthritis; FCM: ferric carboxymaltose; RS3PE: remitting seronegative symmetrical synovitis with pitting edema

Case	Age/sex	Initial suspicion	Final diagnosis	Key diagnostic clue
1	55 F	RA flare	FCM-induced osteomalacia	Hypophosphatemia
2	55 F	RA	Calcific tendinitis	Shoulder calcification
3	45 M	RA	Polyarticular gout	Hyperuricemia
4	68 F	RA	RS3PE with malignancy	Pitting edema
5	76 M	RA flare	Carcinomatous polyarthritis	Colon adenocarcinoma

Across the presented cases, several recurring clinical red flags were identified that may help differentiate RA from alternative diagnoses in routine clinical practice. These distinguishing features are summarized in Table 3.

**Table 3:** Diagnostic red flags for RA mimickers. These features should be interpreted as diagnostic red flags rather than disease-specific findings, as they may overlap with or coexist with RA. - indicates data not available or not applicable. RA: rheumatoid arthritis; DIP: distal interphalangeal

Feature	Suggests RA	Suggests alternative diagnosis
Persistent synovitis	✔	-
DIP involvement	-	✔
Pitting edema	-	✔
Weight loss	-	✔
Hypophosphatemia	-	✔
Hyperuricemia	-	✔

## Discussion

This case series highlights a spectrum of clinically important RA mimickers encountered in real-world rheumatology practice, emphasizing the importance of careful diagnostic evaluation. The cases presented in this series represent distinct categories of RA mimickers, including metabolic bone disease, crystal-induced arthropathy, peri-tendinous calcific disease, and paraneoplastic rheumatic syndromes. This spectrum reflects the diverse mechanisms by which non-rheumatoid conditions may present with polyarticular symptoms resembling inflammatory arthritis.

The differential diagnosis of RA remains a major clinical challenge, particularly in patients presenting with early inflammatory arthritis or seronegative disease. Although the 2010 ACR/EULAR classification criteria improved the sensitivity for early diagnosis of RA, these criteria were primarily designed for classification rather than diagnosis and may lead to misclassification in patients with conditions that mimic RA [2,3].

Our case series highlights several important categories of diseases that can mimic RA in clinical practice, including metabolic bone disorders, crystal-induced arthropathies, peri-tendinous calcific diseases, and paraneoplastic rheumatic syndromes. One important category of RA mimickers includes crystal-induced arthropathies, particularly gout. While gout classically presents as acute monoarthritis affecting the first metatarsophalangeal joint, polyarticular involvement may occur in long-standing disease and can closely resemble RA. Chronic gout may involve small joints of the hands and can produce erosive changes that mimic RA radiographically. Hyperuricemia and response to colchicine may provide supportive clinical clues but are neither specific nor diagnostic of gout. When feasible, identification of monosodium urate crystals in synovial fluid or tophaceous material remains the definitive diagnostic approach [8,11].

Another important RA mimicker is hydroxyapatite crystal deposition disease, which may manifest as calcific tendinitis. Acute episodes of calcific tendinitis may produce severe pain, limited joint movement, and inflammatory features that can resemble septic arthritis or inflammatory arthritis. Radiographic identification of calcific deposits around tendons remains a key diagnostic feature [9].

Metabolic bone disorders such as hypophosphatemic osteomalacia represent another diagnostic pitfall. Ferric carboxymaltose therapy has recently been recognized as a cause of FGF-23-mediated renal phosphate wasting leading to osteomalacia. Patients typically present with diffuse bone pain, muscle weakness, and multiple insufficiency fractures. These symptoms may be mistaken for inflammatory arthritis or fibromyalgia, particularly in patients with underlying rheumatic diseases [6,7].

Paraneoplastic rheumatic syndromes also represent important mimickers of inflammatory arthritis. Carcinomatous polyarthritis is a rare paraneoplastic syndrome characterized by sudden-onset polyarthritis that may precede the diagnosis of malignancy. Similarly, RS3PE syndrome has been associated with a variety of malignancies and should prompt appropriate malignancy screening, particularly in elderly patients presenting with systemic symptoms such as weight loss [4,5,10].

Several clinical features may raise suspicion for an alternative diagnosis rather than RA. These include atypical joint distribution, absence of persistent synovitis, discordance between clinical findings and inflammatory markers, unexplained systemic symptoms, and unusual laboratory abnormalities such as hypophosphatemia or hyperuricemia. Awareness of these diagnostic clues may help clinicians avoid misdiagnosis and unnecessary immunosuppressive therapy [3,12].

**Figure 6:**
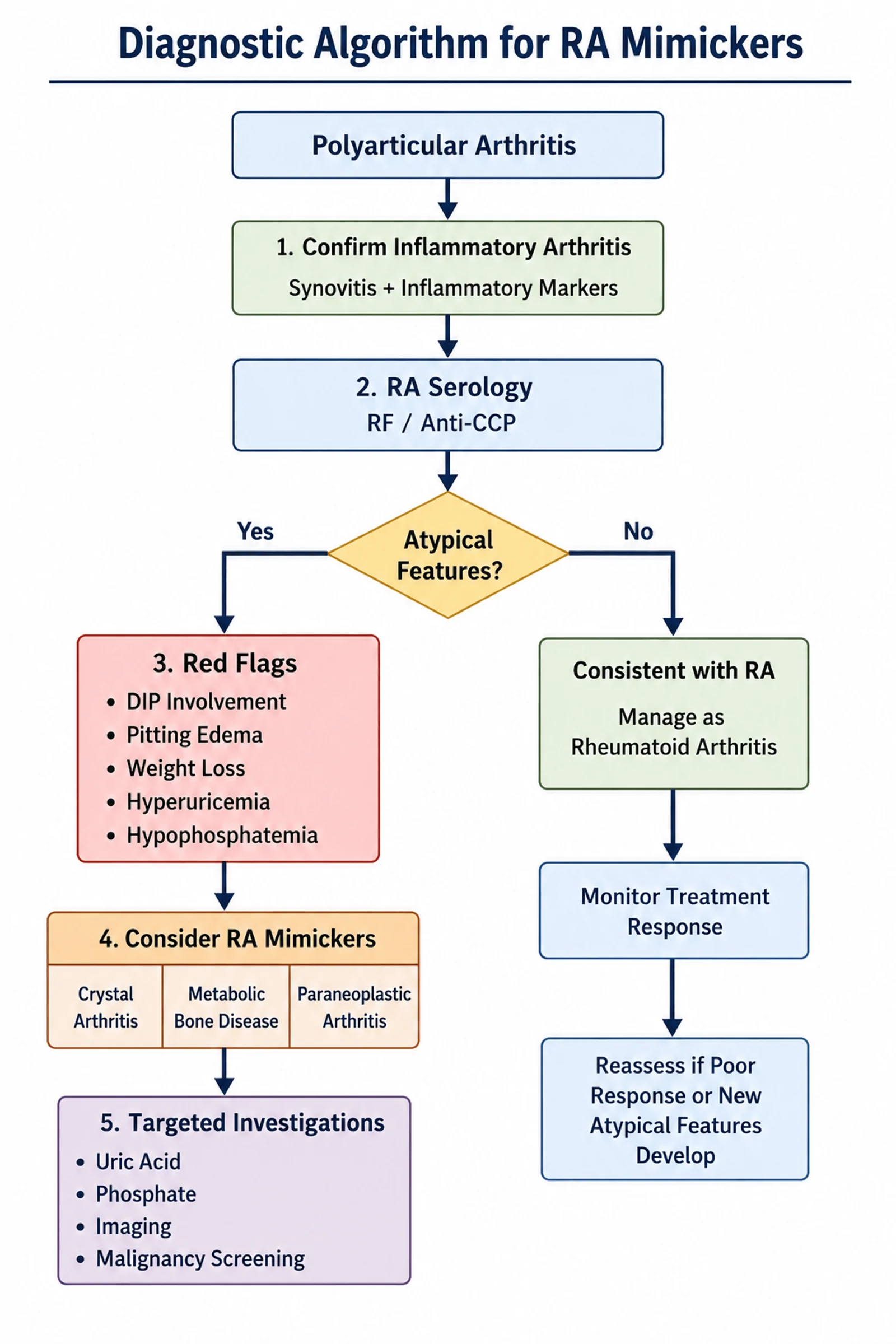
Diagnostic approach to RA mimickers. RA: rheumatoid arthritis; RF: rheumatoid factor; anti-CCP: anti-cyclic citrullinated peptide; DIP: distal interphalangeal

Recognition of these diagnostic clues may help guide a structured diagnostic approach. Based on the clinical observations in this series, we propose a practical diagnostic approach to patients presenting with polyarticular arthritis and atypical clinical features (Figure 6).

This algorithm emphasizes recognition of clinical red flags that should prompt consideration of alternative diagnoses rather than immediate classification as RA. Our case series highlights the importance of maintaining a broad differential diagnosis when evaluating patients with suspected RA. Careful clinical evaluation combined with targeted laboratory and imaging investigations remains essential to identify RA mimickers and ensure appropriate patient management.

Key distinguishing features between RA and its mimickers are summarized in Table 4.

**Table 4:** Key clinical features distinguishing rheumatoid arthritis (RA) from its mimickers. Developed from a review of the literature [3-6,8-12]. MCP: metacarpophalangeal; PIP: proximal interphalangeal; DIP: distal interphalangeal; DMARD: disease-modifying antirheumatic drug

Feature	RA	RA mimickers
Onset	Gradual	Often acute or atypical
Joint distribution	MCP, PIP, wrists	Variable (shoulder, DIP, asymmetric)
Synovitis	Persistent	Often absent or mild
Inflammatory markers	Elevated	May be normal or disproportionate
Systemic symptoms	Fatigue, stiffness	Weight loss, edema, malignancy signs
Radiology	Marginal erosions	Calcifications, metabolic changes
Response to DMARDs	Good	Poor

## Conclusions

RA mimickers represent an important diagnostic challenge in clinical practice. Metabolic bone diseases, crystal-induced arthropathies, calcific tendinitis, and paraneoplastic syndromes may closely resemble RA and lead to diagnostic delay or inappropriate immunosuppressive therapy. Careful clinical assessment, targeted laboratory investigations, and appropriate imaging are essential to identify diagnostic red flags and establish the correct diagnosis, ultimately improving patient management.
